# The Utility of a Plain Film Arthrogram to Confirm Acute Liner Dissociation in the Setting of Primary Total Hip Arthroplasty

**DOI:** 10.7759/cureus.9951

**Published:** 2020-08-23

**Authors:** Paula McQuail, Peggy E Miller, Patrick Nolan, Prasad Ellanti, Tom McCarthy

**Affiliations:** 1 Trauma and Orthopaedic Surgery, St. James's Hospital, Dublin, IRL; 2 Trauma and Orthopaedic Surgery, St. Vincent's University Hospital, Dublin, IRL

**Keywords:** liner dissociation, orthopaedics trauma, hardware malufunction, arthrogram

## Abstract

Acute Liner dissociation is a well-documented, but uncommon complication of total hip arthroplasty, yet the journey to diagnosis remains undefined. This clinical case report outlines the use of plain film arthrogram for diagnosis in a 53-year-old female who presented to the ED following a fall, describing symptoms of increasing groin pain, reduced range of movement, difficulty weight-bearing and a grinding sensation in her left hip, all on a background of total hip replacement two years ago. Examination revealed impaired flexion, rotation and abduction while AP pelvic X-ray confirmed mild eccentric placement of the femoral head, and lateral X-ray proved joint enlocation. An arthrogram of the left hip was performed the following day with injection of 4mls of iodinated contrast injected into the joint. Inferior dissociation of the liner from the shell was evident. The femoral head and liner were replaced two days later, and the liner was found to have shearing and gross plastic deformation at the rim. The patient reported immediate relief from the groin pain and was discharged on the fourth day postoperatively. This shows how plain film imaging fails in diagnosing acute liner dissociation dynamic fluoroscopic tests, post-arthrography CT and metal artifact reduction sequence magnetic resonance imaging (MARS MRI) have previously been proposed despite their associated wait-time, radiation exposure and financial costs. This case report highlights the role of plain film arthrography as a low risk and low-cost diagnostic tool. The report also suggests the incorporation of radio-dense markers in liners to facilitate the use of arthrography when diagnosing dissociation, also raising awareness of prevention and recognition in what may be an under-reported complication of hip arthroplasty.

## Introduction

Total hip arthroplasty liner dissociation is a diagnosis requiring incisive clinical expertise to differentiate prosthetic hip dislocation from catastrophic implant wear or failure. Liner dissociation has been documented in the literature with various methods of diagnosis being proposed from dynamic fluoroscopy, arthrography and post-arthrography computed tomography (CT), double-contrast CT and metal artifact reduction sequence magnetic resonance imaging (MARS MRI) [1,2].

The infrequent reports of liner dissociation in the literature have documented varying incidence rates from 0.046% to 0.8%. These varied statistics are from both institutional reports and national joint registries [3-5]. It has been proposed that the true incidence may yet remain underreported and therefore this complication may be more common than we believe [6]. The apparent infrequency of this complication can result in missed or delayed diagnosis with resultant loosening or destruction of the acetabular cup and or a metallosis reaction necessitating a larger revision procedure than that of early liner dissociation recognition and revision.

This case report serves to stress the importance of retaining clinical suspicion of liner dissociation in a patient complaining of audible hip ‘grinding’ combined with plain film radiographs that may be misinterpreted as a hip dislocation. Furthermore we sought to document the utility of plain radiograph arthrography to confirm the diagnosis.

## Case presentation

A 53-year-old female presented to the ED following a fall while walking, complaining of increasing difficulty weight-bearing due to groin pain and a dramatic reduction in her range of movement of her left hip. Her pain had been present for approximately six weeks but was gradually worsening, particularly with weight-bearing. She described a sensation of grinding in her left hip with intermittent audible ‘squeaking’. She had a left total hip arthroplasty (THA) performed two years ago and described intermittent left groin pain prior to this event for three months. Her primary THA was for hip osteoarthritis and the components used were an uncemented Trilock femoral stem (Warsaw, IN), a Pinnacle 52mm (Warsaw, IN) multihole hemispherical acetabular shell and a 52/32mm highly cross-linked polyethylene Marathon liner (Warsaw, IN).

**Figure 1:**
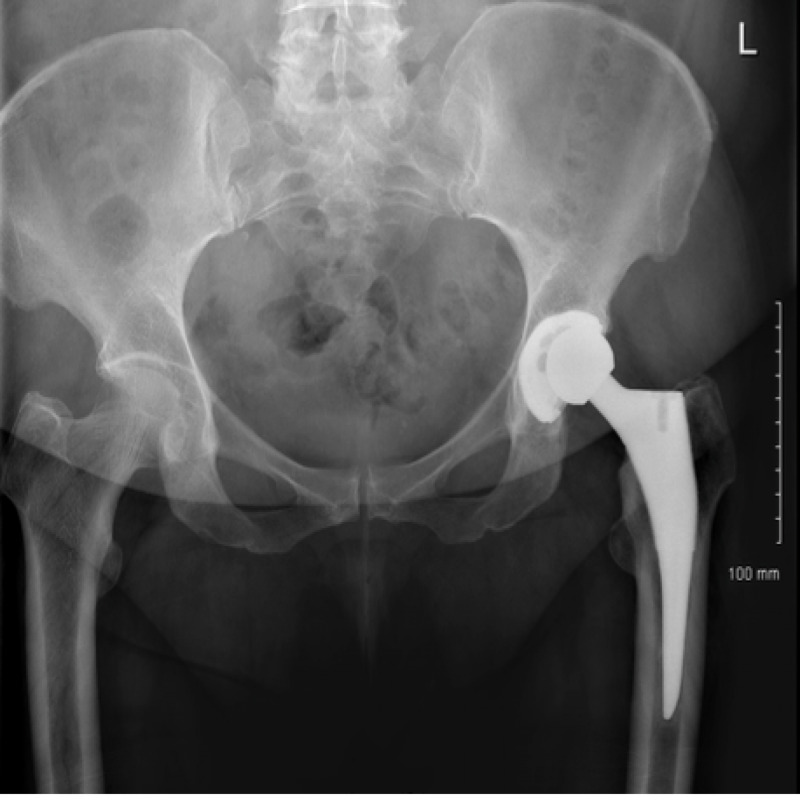
AP view of left hip on presentation demonstrating eccentric femoral head placement with the uncemented acetabular component

**Figure 2:**
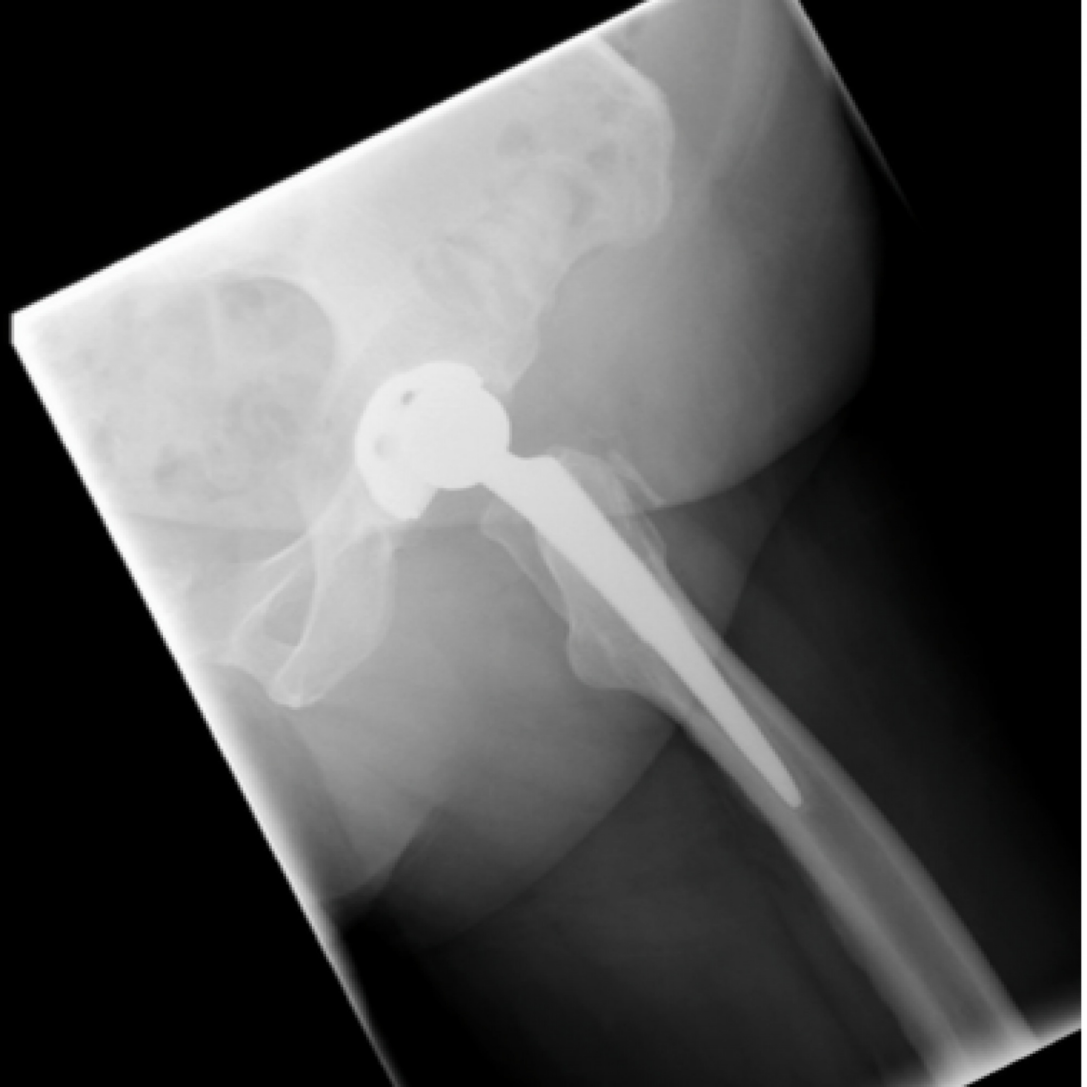
Lateral view of left hip on presentation

The ED physician referred the patient to the orthopaedic service on call as a posteriorly dislocated left total hip replacement. An isolated antero-posterior (AP) pelvic X-ray was obtained on referral which showed mild eccentric placement of the femoral head within the acetabular shell (Figure 1). The tangential lateral x-ray requested by the orthopaedic team confirmed joint enlocation; however, with the femoral head situated eccentrically within the acetabular cup (Figure 2). 

Clinical examination of the patient revealed limited flexion, internal and external rotation and abduction of 40, 20, 20,20 degrees, respectively. Significant groin discomfort was present with passive hip movements. There was audible grinding with passive movement of the hip. The attitude of the hip was not adducted or internally rotated. No significant limb length discrepancy was noted. She had no medical co-morbidities and was a low demand patient with normal body mass index. Differential diagnosis was that of a fracture of the ceramic head, catastrophic liner wear, deep infection or liner dissociation. 

**Figure 3:**
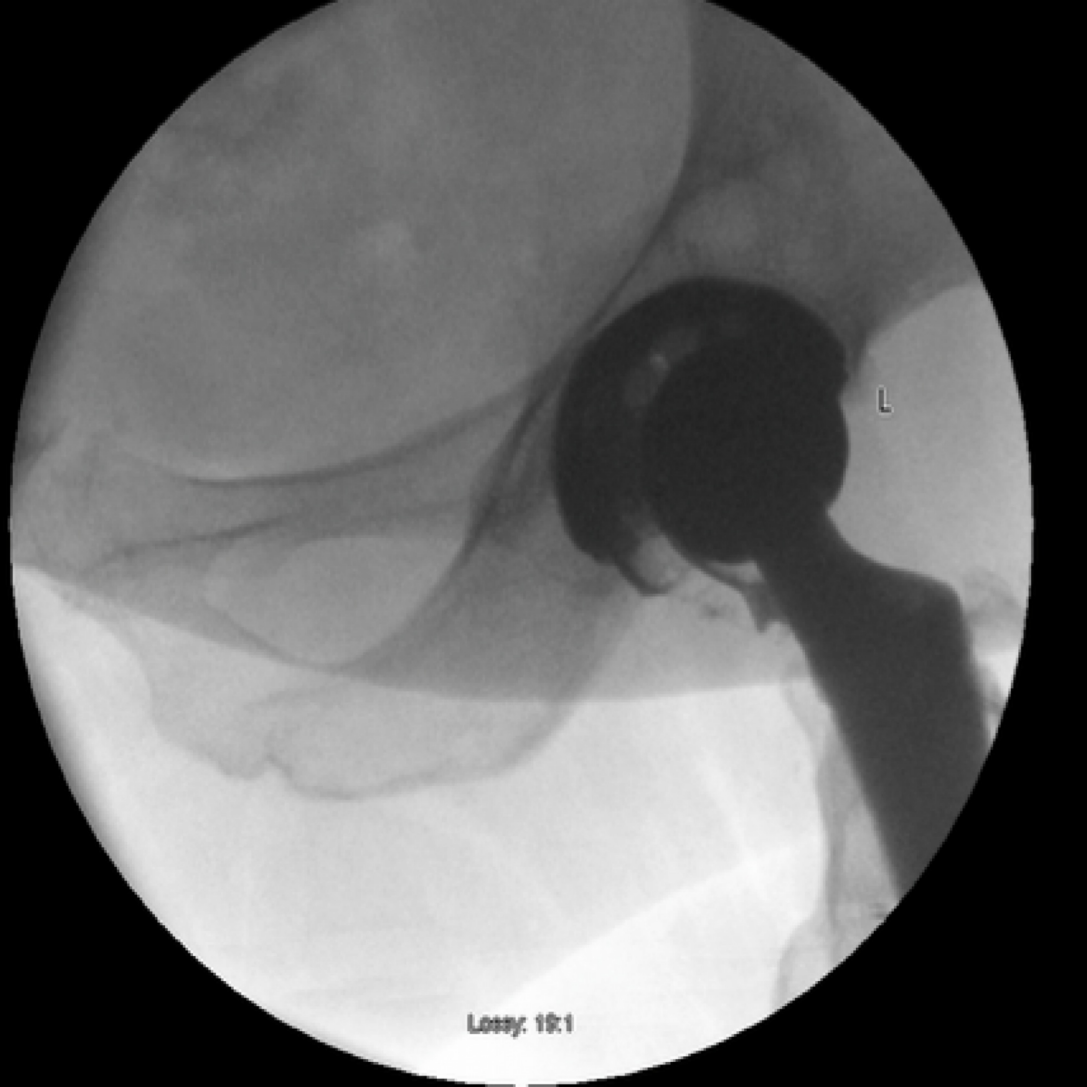
Left hip arthrogram demonstrating an intact but inferiorly dissociated liner and eccentric placement of the femoral head within the acetabular shell

**Figure 4:**
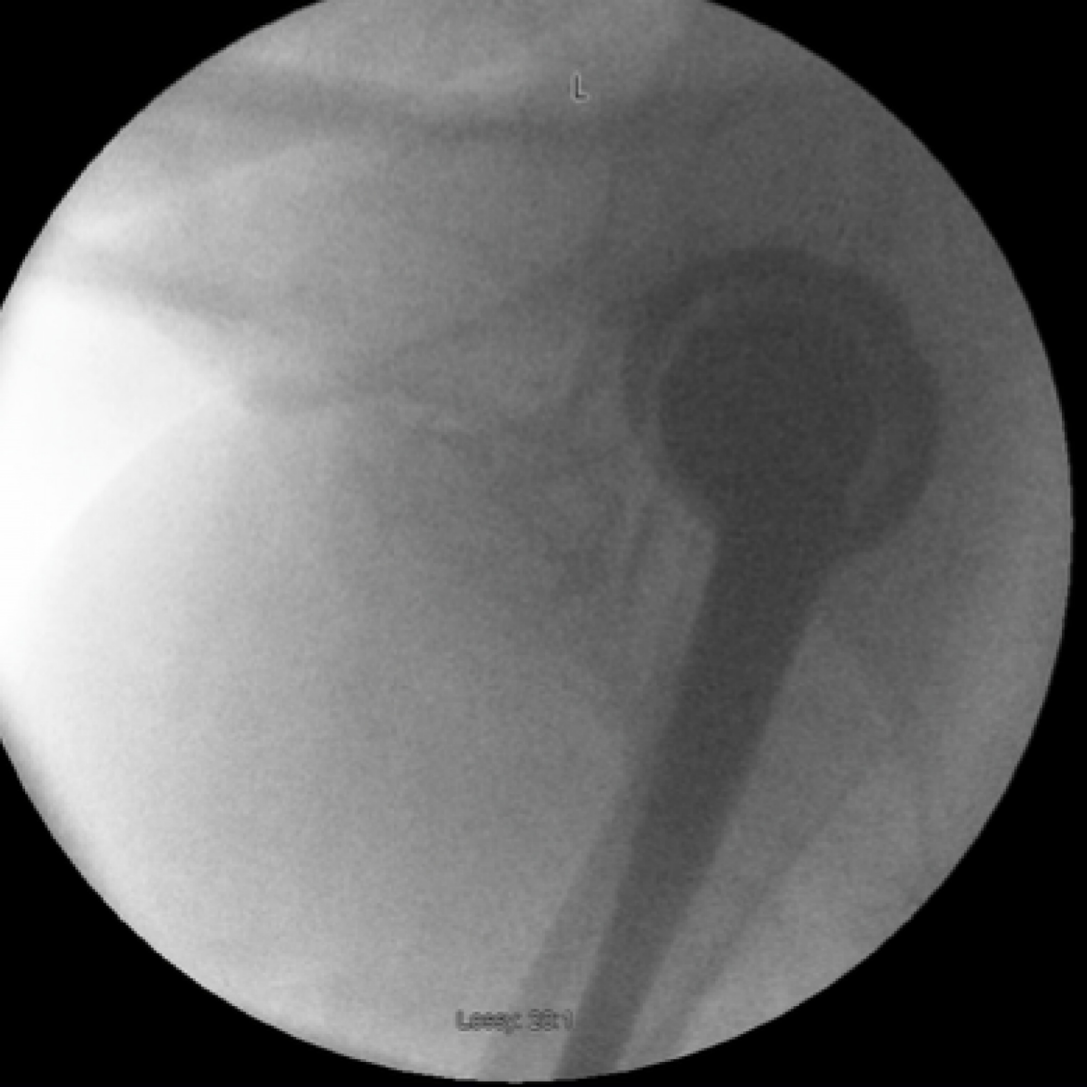
Left hip arthrogram

An arthrogram of her left hip was performed the following day in theatre under local anaesthesia. A 20-gauge spinal needle was inserted into the left hip joint under x-ray guidance. Joint fluid was aspirated to outrule infection and 4 mls of iodinated contrast was injected into the joint. This confirmed needle placement within the joint and femoral head enlocation within the acetabular cup. It delineated integrity but inferior dissociation of the polyethylene liner from the Pinnacle acetabular cup component (Figures 3, 4).

 

Live screening of the left hip also confirmed joint enlocation with liner dissociation. In addition, no radiolucencies were detected around the acetabular or femoral components.

The patient subsequently underwent a planned revision two days later following confirmation that the joint fluid culture was negative for infection. The femoral head and liner were exchanged through the previous incision via an anterolateral approach to the hip. The acetabular cup component had no evidence of loosening, nor did the implanted femoral stem. They were not revised as component integrity, stability and positioning were acceptable. The taperlock mechanism within the shell was also grossly unaltered.

**Figure 5:**
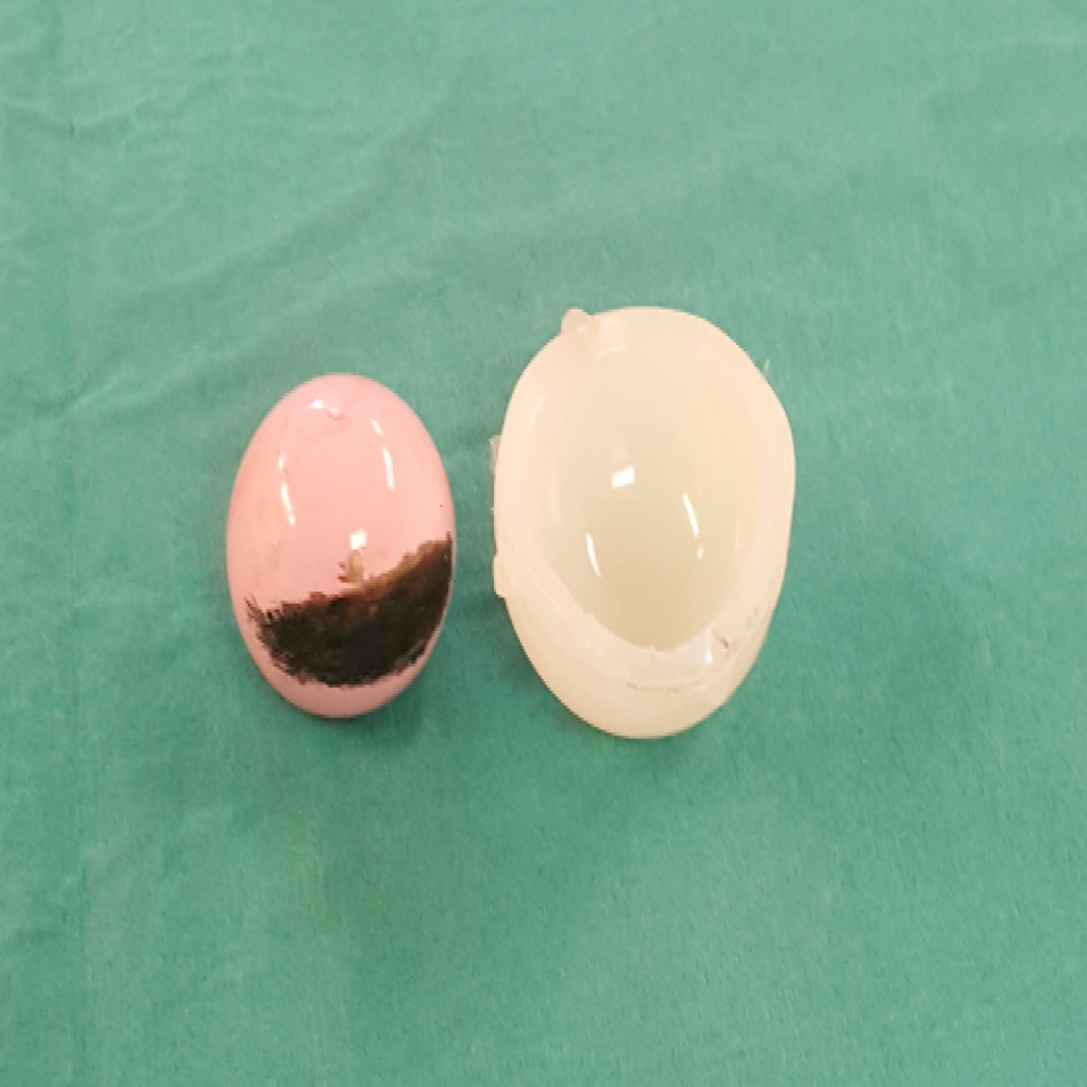
Explanted acetabular cup with marked stripe wear seen as dark discolouration of pink ceramic head and deformed explanted acetabular liner

Intra-operatively the liner was found to be dissociated from the acetabular cup and displaced inferiorly. The explanted ceramic size 32mm head demonstrated dramatic evidence of stripe wear due to edge loading on the implanted acetabular cup and liner rim (Figure 5).

**Figure 6:**
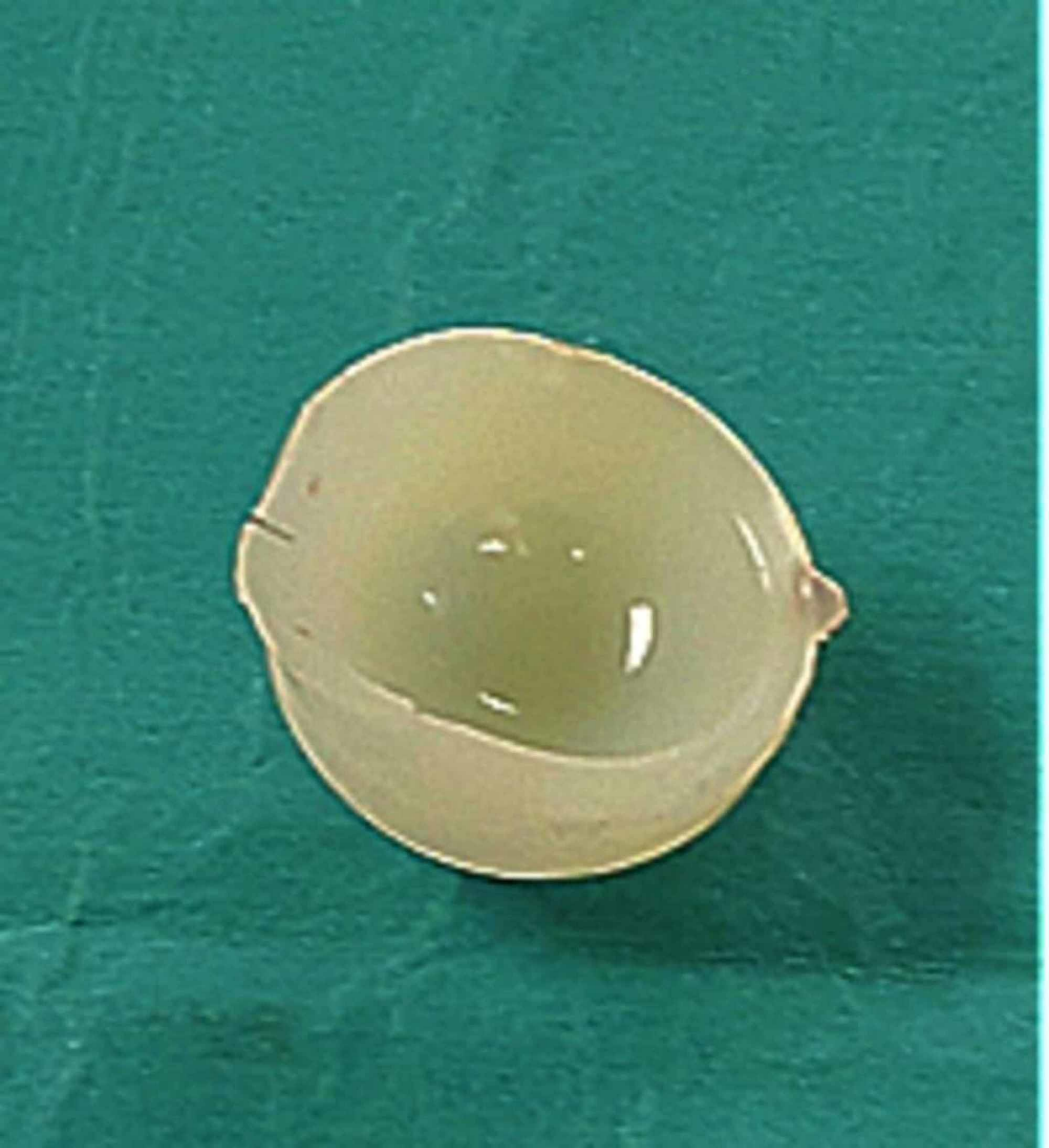
Explanted dissociated polyethylene liner with marked plastic deformation of the rim, new oval shape and multiple tine loss

The Pinnacle shell design incorporates both a taperlock mechanism to secure the liner within it, in addition to multiple anti-rotation device crescentic concavities that accept the liner tines, increasing the overall stability of the liner within the shell. Our patient’s explanted polyethylene liner had evidence of shearing of all but two peripheral anti-rotational locking tines in addition to gross plastic deformation of the rim predominantly at the 1-4 o’ clock position (Figure 6). 

**Figure 7:**
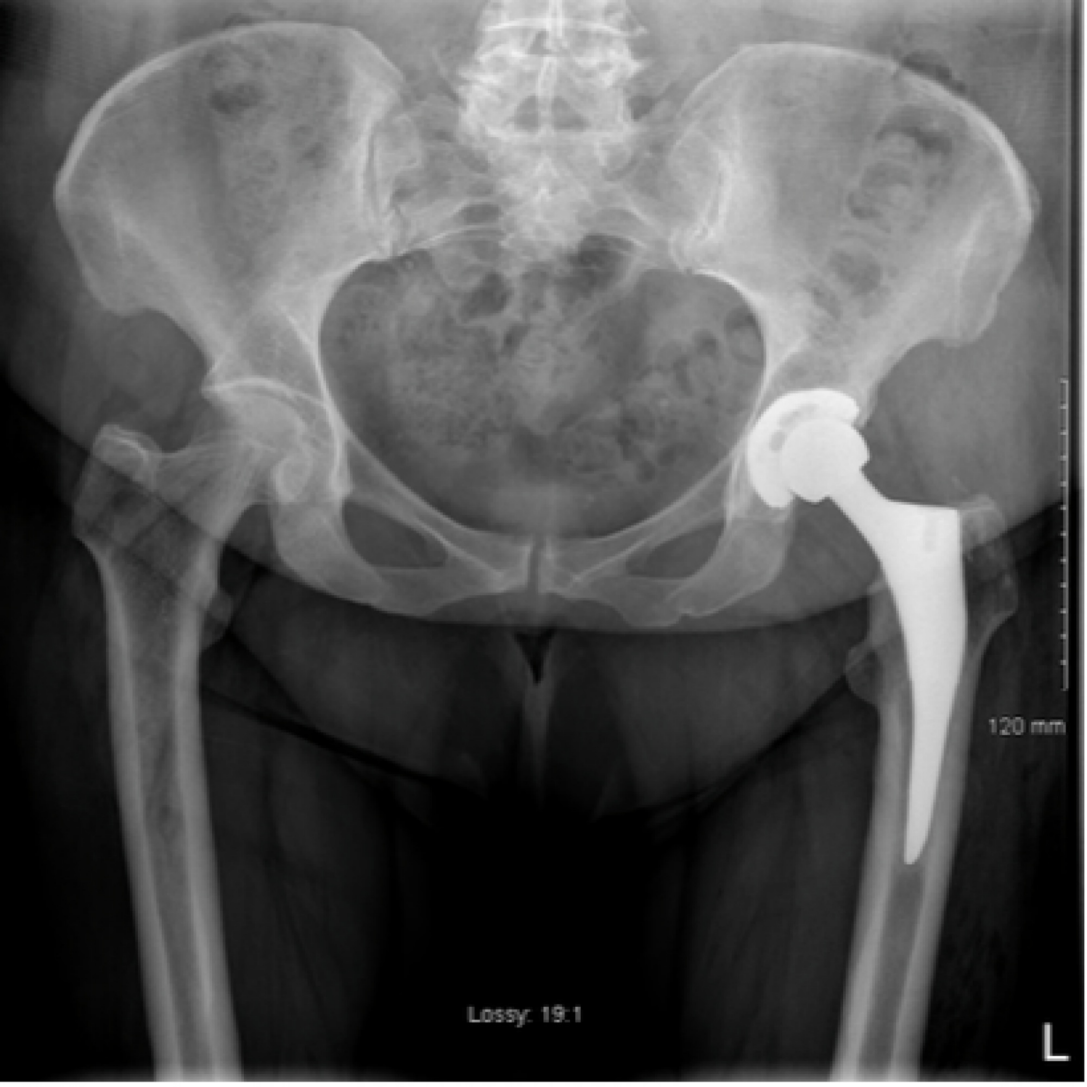
Post-operative AP radiograph of her left hip post head and liner exchange with concentric placement of the femoral head in the acetabular cup

**Figure 8:**
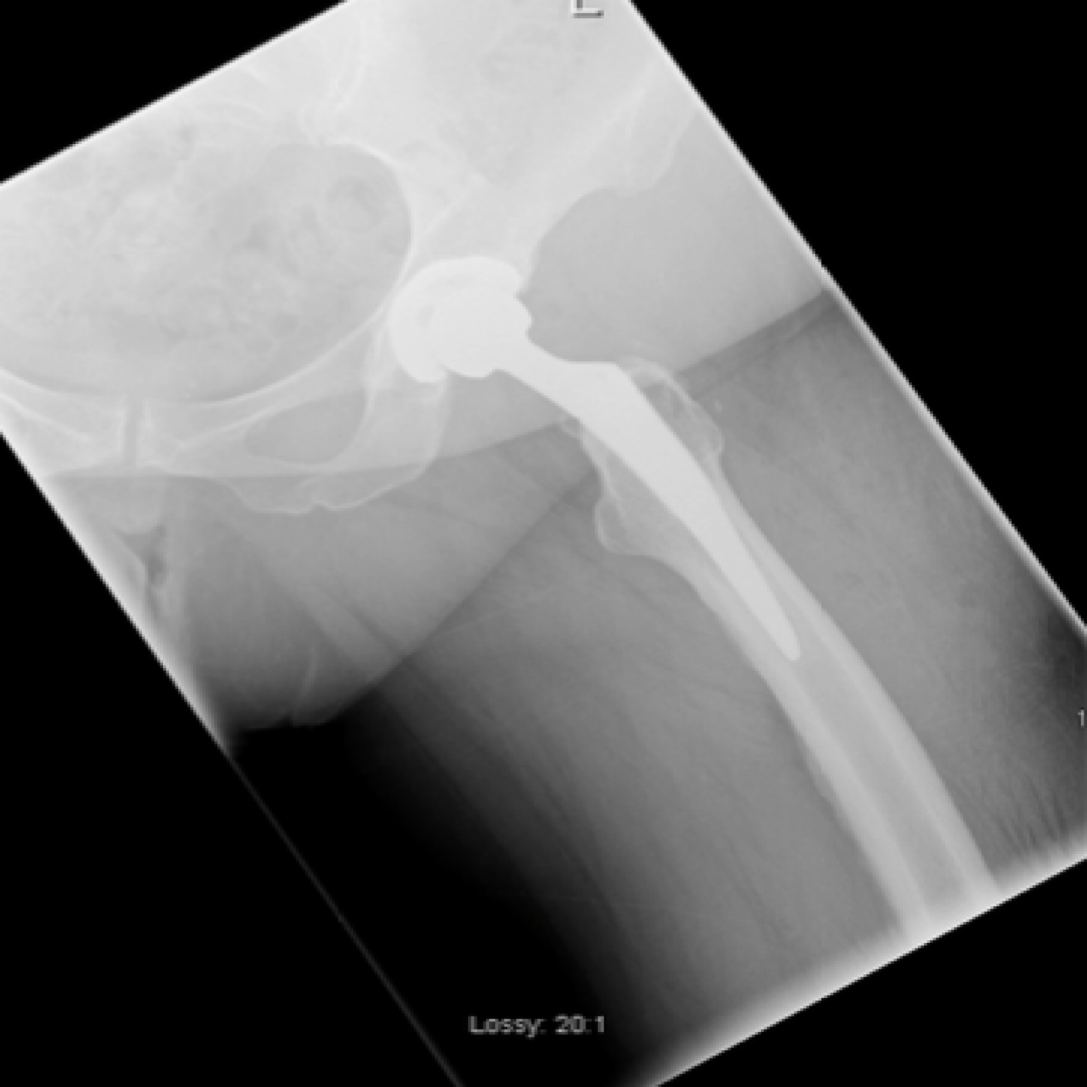
Post-operative lateral radiograph of her left hip post head and liner exchange with concentric placement of the femoral head in the acetabular cup

A new polyethylene liner was successfully seated in the acetabular cup, and a ceramic head was press fit onto the trunnion of the retained femoral component. The revision components were stable on testing with excellent impingement free range of motion preserved. Post-operatively, the patient had immediate relief of the pre-operative groin pain and underwent an uncomplicated post-operative recovery. She was discharged home on the fourth post-operative day. Her post-operative radiograph is seen in Figure 7 and Figure 8.

## Discussion

Acetabular liner dissociation is a serious but uncommon complication following total hip arthroplasty (THA), pertinent to modern modular hip replacement systems. The potential for liner dissociation exists with all modular acetabular cup-liner couplings of THA. However, the literature suggests that this rare complication may be more common with Pinnacle cups and the older Harris Galante 1 and 2 designs [3,7-10]. It appears to be more commonly associated with polyethylene liners due to a differing design from the ceramic liner locking mechanism [11]. The United Kingdom (UK) National Joint Registry (NJR) collated data on 77,009 hips in September 2016 demonstrating a crude revision rate of 0.055% for liner dissociation. The UK NJR have shown a lower rate of revision for dissociation with the use of lipped liners and a higher association in obese patients and in the surgeons early period of experience of use with a Pinnacle cup (De Puy, Warsaw, IN) [12]. 

Early and late liner dissociation are described in the literature [3,7,12-14]. It is possible that early liner dissociation may be related to poorly seated components rather than fatigue failure of the locking mechanism. The senior author’s routine practice is to check that the locking mechanism is stable and completely seated at the time of initial implantation by inspecting the entire acetabular component rim and its interdigitation with the liner. 

The most likely precipitating event for liner dissociation in our patient was the failure of the locking mechanism of the polyethylene liner, resulting in direct contact between the ceramic head and metal acetabular cup and mode two wear. The precise failure cause of the locking mechanism, however, is difficult to determine. It may be related to a defect in the material, fatigue failure of the material, poor implantation technique, inaccurate component positioning, prominent screw heads within the acetabular cup or from an acute hip dislocation where the liner rotated and the sharp edge of the metal cup severs the tines during dislocation [2,15].

Finite element analysis of the DePuy Pinnacle (De Puy, Warsaw, IN) has demonstrated previously that the push and lever-out strength of their liner is weaker than previous designs. Our patient’s complication inciting factor remains uncertain but may be a clinical result of this finding [11].

Although liner dissociation is a rare complication following total hip arthroplasty, a high level of suspicion is required for the diagnosis of liner dissociation to avoid misdiagnosis and to facilitate early appropriate intervention. Clinical presentation findings such as groin pain in a patient with a THA and an associated audible clicking or grinding noise is characteristic [16]. Prosthetic hip dislocation is a differential diagnosis, necessitating astute clinical acumen to differentiate this from liner dissociation. Clinical and radiographic findings can be misinterpreted for hip dislocation [17]. Inferior liner displacement following liner dissociation results in asymmetric placement of the femoral head in the acetabular component, apparent on plain radiograph. The ‘crescent sign’ has been documented in the literature following a review of nine cases of dissociation of modular cups [18]. This sign appears as a crescent-shaped radiolucency medial to the femoral head on the anteroposterior radiograph of the pelvis, with eccentric placement of the head. However, this sign can be insufficient in isolation for diagnosis polyethylene liner dissociation and could represent catastrophic implant wear or failure of the liner, or indeed posterior dislocation of the hip. A false-positive rate of up to 22% has been reported with this sign [19]. 

Dynamic fluoroscopic tests, arthrography and post-arthrography computed tomography (CT), double-contrast CT arthrography and MARS MRI have been proposed in instances where plain film imaging fails to yield a definitive diagnosis (1-4). CT and MRI, however, have incumbent diagnostic limitations due to metal artifact created by the hip arthroplasty implant, in addition to the expense of both modalities. These imaging modalities are often overburdened in the public hospital setting adding further difficulty for clinicians seeking to obtain a timely diagnosis and treatment. Jang et al. reported a mean period between pelvic radiography and CT as 3.1 days (range 0-16) and a period mean of 23.0 days (range 1-98 days) from pelvic radiography to revision surgery [19]. CT exposes the patient to considerably more radiation than plain radiographs which underlines the importance of appreciating the utility of a plain film hip arthrogram as a diagnostic tool. Dynamic fluoroscopy post arthrogram can help confirm the diagnosis as we outlined. The surgeon can also use the opportunity to aspirate the hip to out rule infection at the time which will is useful prior to planning a revision procedure.

We understand that CT and MARS MRI are useful diagnostic modalities. However, we advocate their use as second-line investigations if plain film fails to confirm the diagnosis, once liner dissociation is suspected from the pelvic plain film X-ray. To our knowledge, the role of plain film arthrography as a diagnostic, low radiation, accessible and cost-efficient tool also facilitating joint aspiration for pre-operative workup has not been documented to date.

Although the United Kingdom and Australian registry data demonstrates survivorship of the DePuy Pinnacle acetabular cup system comparable with other implants, it is prudent for clinicians to be aware that liner dissociation is a complication documented in the literature somewhat more frequently than with other modular implant systems [3,10,12,13,17]. It remains, however, a rare complication and this prosthesis has an excellent track record among registry data historically.

To minimise liner dissociation as a post-operative complication, the surgeon must pay attention to accurate component positioning, and implantation, ensure the taper lock device and tines are well seated when the liner is impacted into the cup, with no interfacial soft tissue present, and counsel the patient regarding their positioning precautions in the post-operative period. The surgeon’s choice of components is another variable which may be altered. Modular hip arthroplasty components whereby the liner is cemented in place are less frequented as a choice but are commercially available. Biomechanical studies indicate that the strength of fixation is greater with this method than in some conventional commercially available cementless standard locking mechanisms [20]. However, dissociation of a cemented liner from failure at the cement liner interface has also been reported in clinical studies and is not a failsafe to prevent this complication [18]. This appears to have been related to the cementation of constrained liners or cementation of a liner with a greater diameter than the retained cup diameter.

## Conclusions

In conclusion, this paper characterises the utility of plain film arthrography as a simple accessible and cost-efficient tool for the diagnosis of liner dissociation. We would support the incorporation of radio-dense markers within the polyethylene component to facilitate a simple and reliable plain x-ray diagnosis of such a complication post THA. We believe this paper will raise awareness and potentially prevent and recognize what may be an under-reported failure mechanism following hip arthroplasty.
